# The Post Clinic Ambulatory Blood Pressure (PC-ABP) study correlates Post Clinic Blood Pressure (PCBP) with the gold standard Ambulatory Blood Pressure

**DOI:** 10.1186/s13104-018-3509-0

**Published:** 2018-07-11

**Authors:** Hunaina Shahab, Hamza Sohail Khan, Aysha Almas, Mayera Tufail, Khawar Abbas Kazmi, Aamir Hameed Khan

**Affiliations:** 1 Cardiology, Aga Khan University Hospital, Second Floor, Faculty Offices Building, Stadium Road, P.O. Box 3500, Karachi, 74800 Pakistan; 2 Internal Medicine, Aga Khan University Hospital, Second Floor, Faculty Offices Building, Stadium Road, P.O. Box 3500, Karachi, 74800 Pakistan

**Keywords:** Post-clinic blood pressure, Ambulatory Blood Pressure Monitoring, White-coat effect

## Abstract

**Objective:**

Our previous study showed that post-clinic blood pressure (BP) taken 15 min after a physician–patient encounter was the lowest reading in a routine clinic. We aimed to validate this reading with 24 h Ambulatory Blood Pressure Monitoring (ABPM) readings. A cross-sectional study was conducted in the cardiology clinics at the Aga Khan University, Pakistan. Hypertensive patients aged ≥ 18 years, or those referred for the diagnosis of hypertension were included.

**Results:**

Of 150 participants, 49% were males. 76% of all participants were hypertensive. Pre-clinic BP reading was measured by a nurse, in-clinic by a physician and 15 min post-clinic by a research assistant using a validated, automated BP device (Omron-HEM7221-E). All patients were referred for 24 h ABPM. Among the three readings taken during a clinic visit, mean (± SD) systolic BP (SBP) pre-clinic, in-clinic, and 15 min post-clinic were 153.2 ± 23, 152.3 ± 21, and 140.0 ± 18 mmHg, respectively. Mean (± SD) diastolic BP (DBP) taken pre-clinic, in-clinic and 15 min post-clinic were 83.5 ± 12, 90.9 ± 12, and 86.4 ± 11 mmHg respectively. Mean (± SD) daytime ambulatory SBP, DBP and pulse readings were 134.7 ± 15, 78.7 ± 15 mmHg, and 72.6 ± 12/min, respectively. Pearson correlation coefficients of pre-clinic, in-clinic and post-clinic SBP with daytime ambulatory-SBP were 0.4 (p value: < 0.001), 0.5 (p value: < 0.001) and 0.6 (p value: < 0.001), respectively. Post-clinic BP has a good correlation with ambulatory BP and may be considered a more reliable reading in the clinic setting.

## Introduction

Clinic blood pressure (BP) does not correlate well with 24 h Ambulatory Blood Pressure Monitoring (ABPM) [1], and is unable to overcome white-coat effect [2]. National Institute for Health and Clinical Excellence in the United Kingdom has recommended the use of ABPM to confirm the diagnosis of hypertension [3]. However, the test is expensive [2] and in a developing country like Pakistan, many patients are unable to afford ABPM. It is also cumbersome, intolerable to a few patients, particularly during sleep [4], and requires expertise for analysis [5]. Therefore it was important to investigate which BP reading measured in clinic came closest to the ABP reading.

Our preliminary work showed that the post-clinic systolic BP (SBP) reading taken 15 min after the physician’s encounter was 10 mmHg lower than the SBP reading taken in the clinic (p value < 0.001) [6]. We, therefore, conducted this study aiming to validate post-clinic BP (SBP and DBP) taken 15 min after the physician–patient encounter with 24 h ABPM.

## Main text

### Methods

#### Study site, population, and definitions

A cross-sectional study was conducted in the outpatient cardiology clinics at the Aga Khan University Hospital, Pakistan, over a year and a half period starting 2015. Patients who were either hypertensive (defined as those with a clinic SBP ≥ 140 mmHg or DBP ≥ 90 mmHg [7]) or referred for the assessment of hypertension, aged ≥ 18 years were recruited. Pregnant females, those with a history of volume loss or taking NSAIDs were excluded. White-coat hypertension was defined as clinic BP ≥ 140/90 mmHg with average ambulatory daytime BP < 135/85 mmHg [8]. White-coat effect was defined as clinic BP ≥ 20/10 mmHg higher than ambulatory BP [9]. Masked hypertension was defined as having a clinic BP < 140/90 mmHg and a daytime ABP ≥ 135/85 mmHg [10].

#### Clinic blood pressure measurement

BP and pulse readings were taken at three points in the clinic. The first BP and pulse reading of each participant was taken by the assessment nurse before the patient–physician encounter as part of the clinic protocol i.e. pre-clinic BP and pulse, after a waiting period (14 ± 1.2 min). The second reading was taken by the attending physician as part of the regular physical examination i.e. in-clinic BP and pulse after a waiting period (16 ± 1.4 min). This waiting time was inevitable due to the high patient number in every clinic and was applicable to each participant recruited in the study. Each participant was then asked to wait for 15 min in the regular waiting area (where smoking and exertion was prohibited). They were then called back to another clinic room where the post-clinic BP and pulse reading was taken by a trained research assistant in the absence of the primary physician. An interval of 15 min was chosen to replicate our previous study [6] and based on van der Wel et al’s study which showed that SBP reaches a plateau within the first 15 min in a clinic [2]. The remaining waiting period in the pre- and in-clinic setting matched the 15 min waiting period of the post-clinic reading. At each of these three points, two BP and pulse readings were taken with 2 min interval between them and the average of the two was used in the final analysis.

For each BP reading, the participant was asked to sit with his/her back supported and feet on the floor; arm supported at the heart level; appropriate size cuff was applied (with the bladder covering 80% of the arm) [7]. We used an automated and validated [11] BP device (OMRON HEM 7221-E, M6 Comfort, Omron Healthcare Europe) to take all BP values to avoid all inter-user variability in the readings. At the start of the study, the nurse, physician and research associate were given a refresher training session in the appropriate method of taking BP to ensure that there was no variability in their methods of measuring BP.

#### Ambulatory Blood Pressure Measurement

After the post-clinic BP reading was taken, a 24 h ABPM monitor (SpaceLabs, model: 90217A) was attached to each participant which took BP and pulse readings every half hour during the daytime and every hour during the nighttime. Each patient was given a diary to record their activity throughout the test period. The test was considered valid when ≥ 85% readings were recorded [12].

The participants were asked to return to the clinic the next day after their 24 h of the ABPM were complete. Each participant was given a cash compensation for their travel and logistic expenses. If a discrepancy was detected between the BP taken during the clinic and the ABPM readings, the participant was scheduled for an early follow-up visit. The participants were explained the side-effects of the ABPM test which included sleep disturbances, pain, skin irritation or bruising due to the cuff [13].

#### Statistical analysis

Statistical Package for Social Sciences (SPSS), version 23 was used for analysis. Mean and standard deviation (SD) were used for quantitative variables, and frequency and percentages for categorical variables. For all three measurement modalities, a mean was computed on the basis of all the measurements taken within each session. Pearson correlation coefficient was used to determine the correlation between post-clinic BP (SBP and DBP) and mean SBP and DBP recorded by the ABPM.

#### Sample size

Sample size calculation was based on the observed differences in SBP between the in-clinic readings and 24 h ABPM readings from existing literature. A minimum sample size of 55 patients was required to show a mean difference of 27 mmHg between in-clinic SBP and daytime ABPM record and a minimum sample size of 18 was required to estimate a mean difference of 18 mmHg between in-clinic SBP and nighttime SBP, at an alpha of 5% and a beta of 80%. Assuming also that the mean difference between post-clinic BP (SBP and DBP) and 24 h overall ambulatory BP (SBP and DBP) record with a power of 80% and a significance level of 5%, was 18 mmHg, a minimum sample of 123 patients was required. The sample size was further inflated by 20% up to 150 to account for non-responders. Keeping in account our clinic logistics and cost of conducting the study, a sample of 150 participants was convenient.

### Results

A total of 162 patients were approached to participate in the study. Nine participants refused as they were unable to return the ABPM apparatus next day. Three participants refused to carry the ABPM device to work the next morning. Of the 150 patients who consented to participate, 49% (n = 73) were males and 76% (n = 114) of all participants were hypertensive. The mean age of the participants was 60.3 ± 11.9 years. Of the 73 males, 71% (n = 52) were hypertensive. Of the 77 females, 80% (n = 62) were hypertensive. Two percent (n = 3) of our participants had stage 2 chronic kidney disease, 8% (n = 12) were smokers, 20% (n = 30) had diabetes mellitus and 30% (n = 45) had coronary artery disease. Eleven (7.3%) of the participants reported the ABPM to interfere in their sleep. Five (3.3%) participants found the test uncomfortable. No major side effect was noted that required discontinuation of the process. Of the 114 hypertensive participants, 43 (38%) had white-coat effect. Of the 36 participants who were referred for the assessment of hypertension, 25% (n = 9) had white-coat hypertension. Six percent (n = 9) participants experienced masked hypertension.

**Fig. 1:**
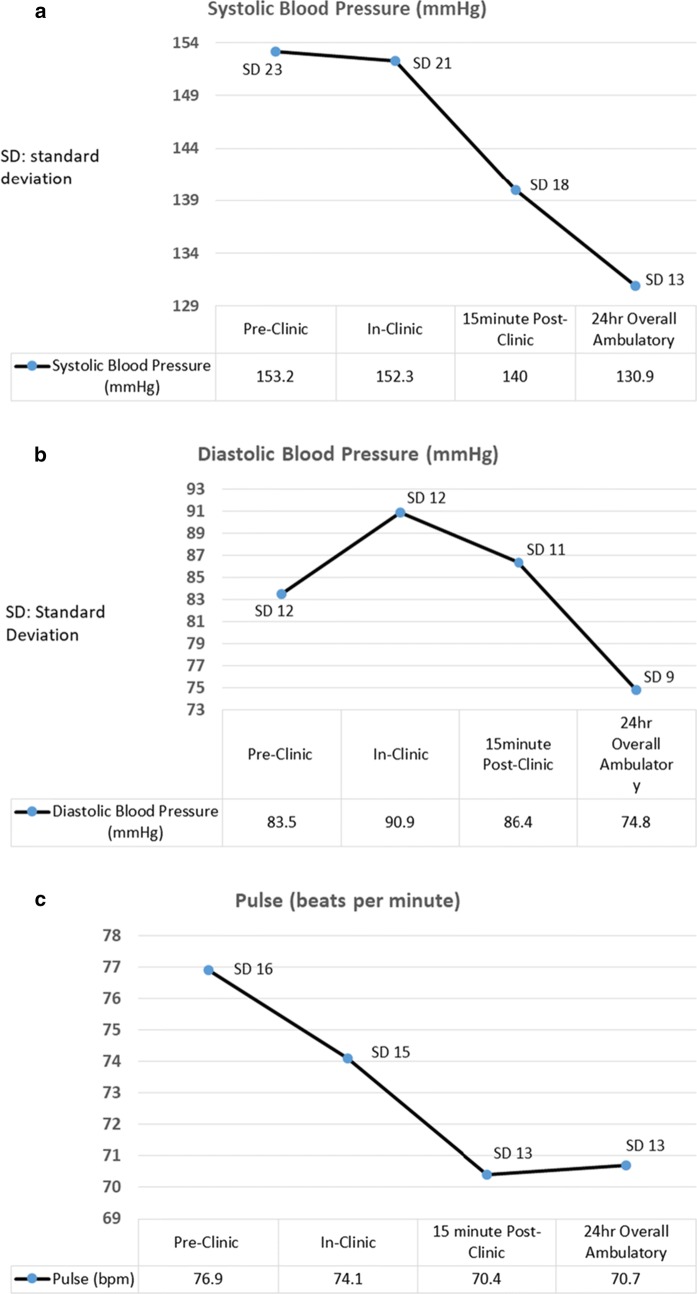
**a** Trends of mean systolic blood pressure (SBP in mmHg) amongst participants, pre-clinic, in-clinic, 15-min post-clinic and 24 h overall ambulatory. **b** Trends in mean diastolic blood pressure (DBP in mmHg) amongst participants, pre-clinic, in-clinic, 15-min post-clinic and 24 h overall ambulatory. **c** Trends in mean pulse values (beats per minute) amongst participants, pre-clinic, in-clinic, 15-min post-clinic and 24 h overall ambulatory

The mean and standard deviation (± SD) SBP taken pre-clinic, in-clinic, 15 min post-clinic and 24 h overall ambulatory SBP is shown in Fig. 1a. The mean and standard deviation (± SD) DBP taken pre-clinic, in-clinic, 15 min post-clinic and 24 h overall ambulatory DBP is shown in Fig. 1b. The mean and standard deviation (± SD) pulse taken pre-clinic, in-clinic, 15 min post-clinic and 24 h overall ambulatory pulse is shown in Fig. 1c.

The mean (± SD) 24 h overall ambulatory SBP, DBP and pulse readings were 130.9 ± 13, 74.8 ± 9 mmHg and 70.7 ± 13/min, respectively. The mean (± SD) daytime ambulatory SBP, DBP and pulse readings were 134.7 ± 15, 78.7 ± 15 mmHg and 72.6 ± 12/min, respectively. The mean (± SD) nighttime ambulatory SBP, DBP and pulse readings were 121.9 ± 18, 68.8 ± 9 and 63.8 ± 10 mmHg.

The Pearson correlation coefficient values of pre-clinic, in-clinic and 15 min post-clinic SBP with 24 h overall ambulatory, daytime ambulatory and nighttime ambulatory SBP are shown in Table 1.

**Table 1:** Correlation of pre-clinic, in-clinic, 15 min post-clinic SBP with ambulatory SBP readings

Pearson correlation coefficient	Pre-clinic SBP	In-clinic SBP	15 min post-clinic SBP
24 h overall ambulatory SBP	0.4	0.5	0.6
	(p value: <0.001)	(p value: < 0.001)	(p value: < 0.001)
Daytime ambulatory SBP	0.4	0.5	0.6
	(p value: <0.001)	(p value: < 0.001)	(p value: < 0.001)
Nighttime ambulatory SBP	0.3	0.4	0.4
	(p value: 0.001)	(p value: < 0.001)	(p value: < 0.001)

The Pearson correlation coefficient values of pre-clinic, in-clinic and 15 min post-clinic DBP with 24 h overall ambulatory, daytime ambulatory and nighttime ambulatory DBP are shown in Table 2.

**Table 2:** Correlation of pre-clinic, in-clinic, 15 min post-clinic DBP with ambulatory DBP readings

Pearson correlation coefficient	Pre-clinic DBP	In-clinic DBP	15 min post-clinic DBP
24 h overall ambulatory DBP	0.4	0.5	0.5
	(p value: < 0.001)	(p value: < 0.001)	(p value: < 0.001)
Daytime ambulatory DBP	0.3	0.3	0.3
	(p value: < 0.001)	(p value: < 0.001)	(p value: < 0.001)
Nighttime ambulatory DBP	0.3	0.4	0.3
	(p value: 0.001)	(p value: 0.000)	(p value: < 0.001)

### Discussion

We found that the BP reading taken 15 min after the clinic ended was the lowest reading of all taken in a real-world clinic encounter and it came closest to the ABPM reading. Post-clinic SBP was about 12 mmHg lower than the reading taken in the presence of a physician, therefore, we think that the readings taken in the post-clinic time can help in alleviating the white-coat effect. Post-clinic DBP was lower than the in-clinic DBP, however, the lowest was the pre-clinic DBP. These findings replicated the results of our previous study [6] therefore our results were noted to be reproducible. Our results were similar to Mancia et al’s study [14] which showed that patients’ BP and heart rate increased when visited by a physician or a nurse, the rise being higher with the physician. Both heart rate and BP declined, over the next 10 min, by about 10/5 mmHg owing to the reduction in the alert reaction [14]. Another study showed that serial automated office BP readings taken in a quiet room using the ABPM device decreased by about 12 mmHg to reach a plateau over 15 min and these readings remained similar at 30 min. This 30 min BP agreed well with the daytime ABPM [2]. Furthermore, our results also followed the same trend as the white coat hypertensive group in Ogedegbe et al’s study [15] which showed that BP and anxiety levels increased in the presence of a physician and then dropped after the physician had left the room, the rise being more dramatic in the white-coat hypertensive population [15].

We also found that the post-clinic BP correlated better with 24 h ambulatory BP and daytime BP as compared to pre-clinic or in-clinic BP. The correlation was stronger for SBP than for DBP. The difference between 24 h ambulatory SBP and post-clinic SBP was the lowest whereas it was the greatest between 24 h ambulatory SBP and in-clinic SBP. Several investigations had attempted to seek an alternative to the 24 h ABPM. 6 and 10 h ABPM was comparable to daytime ABPM [16, 17]. Furthermore, the readings taken by an automated device in the absence of a physician were found to be more comparable to daytime mean ABP readings [18]. However, to the best of our knowledge, the BP readings taken 15 min after the patients’ meeting with the physician had not been compared to 24 h ABPM previously. Since post-clinic BP correlated better with ABPM readings, it may be considered as a surrogate for ABPM.

In clinical practice, office BP is used as a reference for diagnosis [19] and adjustment of antihypertensive medications. Our results highlighted the fact that prescribing medications based on in-clinic or pre-clinic BP readings may result in an undesirable drop in BP. Post-clinic BP can be more reliable than the conventional methods as well as more cost-effective upfront in comparison to ABPM for assessment of hypertension and adjusting medications. It may also be used as an alternate when ABPM or home monitoring is not available.

ABPM, however, is superior to office BP due to its higher prognostic value [20]. It can assess nighttime BP which is a stronger predictor of cardiovascular morbidity and mortality [21]. Further studies with a larger sample size are required to determine the prognostic value of post-clinic BP and its association with cardiovascular outcomes.

### Conclusion

Post-clinic BP is the lowest reading taken in a clinic visit and has a good correlation with ambulatory BP. This reading may be more reliable for assessment of hypertension than in-clinic BP.

## Limitations

- This was a single-centered study conducted only in cardiology clinics limiting generalization in all settings.
- Post-clinic BP is a snapshot value taken during the day hence the dipper/non-dipper status cannot be accounted for by this reading.
- We did not stratify our results according to age, therefore it is possible that different age groups may exhibit variation in results.
- Three different individuals took BP readings which may have an implication on white-coat effect. This was done to replicate a real-world clinic scenario where BP readings are taken by different observers. All readings were taken using the validated automated BP device to minimize inter-observer variability.

## Abbreviations

BP: blood pressure
ABPM: Ambulatory Blood Pressure Monitoring
SD: standard deviation
SBP: systolic blood pressure
DBP: diastolic blood pressure
